# Ultra-Broadband, Lithography-Free, and Large-Scale Compatible Perfect Absorbers: The Optimum Choice of Metal layers in Metal-Insulator Multilayer Stacks

**DOI:** 10.1038/s41598-017-13837-8

**Published:** 2017-11-01

**Authors:** Sina Abedini Dereshgi, Amir Ghobadi, Hodjat Hajian, Bayram Butun, Ekmel Ozbay

**Affiliations:** 1 Department of Electrical and Electronics Engineering, Bilkent University, Ankara, 06800 Turkey; 2 Nanotechnology Research Center (NANOTAM), Bilkent University, Ankara, 06800 Turkey; 3 National Nanotechnology Research Center (UNAM), Bilkent University, Ankara, 06800 Turkey; 4 Department of Physics, Bilkent University, Ankara, 06800 Turkey

## Abstract

We report ultra-broadband perfect absorbers for visible and near-infrared applications that are based on multilayers of metal-insulator (MI) stacks fabricated employing straightforward layer deposition techniques and are, therefore, lithography-free and large-scale compatible. We scrutinize the impact of different physical parameters of an MIMI absorber structure with analysis of each contributing metal layer. After obtaining the optimal design parameters (i.e. material selection and their thicknesses) with both simulation and numerical analysis (Transfer Matrix Method) methods, an experimental sample is fabricated and characterized. Our fabricated MIMI absorber consists of an optically thick tungsten (W) back reflector layer followed by 80 nm aluminum oxide (Al_2_O_3_), 10 nm titanium (Ti), and finally another 80 nm Al_2_O_3_. The experimental results demonstrate over 90 percent absorption between 400 nm and 1640 nm wavelengths that is optimized for ultra-broadband absorption in MIMI structures. Moreover, the impedance matching method with free-space is used to shed light on the metallic layer selection process.

## Introduction

The ongoing trend of scaling down electronic and photonic devices has been leading the research on the track of looking for miniaturized designs and devices. Nanophotonic devices are deemed a hot-topic area and have gained a great deal of attention in decreasing dimensions and increasing functionality. Among the mentioned devices are those that are designed for propagation and guiding, beaming, and confinement of the light. The black body-like absorbers are devices that are capable of almost annihilating reflection and are useful in many applications such as thermal imaging^1^, emitters^2^, photovoltaics^3^, photodetectors^4^, and shielding. Many structures have been proposed for a perfect absorber to satisfy the figures of merit of absorbers that are flat, near unity absorption, high bandwidth, and polarization insensitivity. Conventional bulk absorbers are not efficient, specifically in thin thicknesses, which is a main drive in science and industry. With the advent of plasmonics, many reports were obsessed with plasmonic MIM resonators that amended the functionality of devices compared to conventional bulk absorber semiconductors and gave the flexibility of geometry alterations to tune the bands and bandwidths. Kim *et al*. put forth MIM absorbing stacks while focusing on identifying the bases of localized and non-localized plasmon resonance enhanced absorptions^5^. Aydin *et al*. reported more complicated features for broadband MIM absorbing stacks fabricated using the electron beam lithography (EBL) method; the absorption reached an average amount of 71 percent within the 400 nm to 700 nm wavelength range^6^. In order to increase the bandwidth of absorbers, multi-feature plasmonic cells were also reported^7^; however, the boost of bandwidth comes at the expense of the absorption peak that is a major setback of these designs. Generally, plasmonic absorbers function on the basis of bringing together the peaks of absorption due to confinement of light in nanoresonator-air (excitation of localized surface plasmons) and nanoresonator-insulator (excitation of surface plasmons) media. However, they require the time-consuming and expensive EBL method. There have been reports to tailor lossy metals in MIM plasmonic absorbers that require EBL^8^. Another possible design is using random sized-nanoparticles attained by the dewetting method together with lossy metals^9^. This design still requires high temperature budgets and imposes a dilemmatic trade-off between the bandwidth and peak of absorption and the bandwidths are not competitive enough to outperform cavity-like absorbers. One of the broadest absorptions is reported in pyramid structures composed of metal-dielectric films^10^ that are difficult to fabricate. Another design worth pointing out is using layered tandem cell structures that are reasonably simple while fulfilling figures of merit of absorbers to a large degree^11^. Despite being lithography-free, they require the deposition of many different layers that might be prohibitive and the repeatability and overall throughput is a concern in this design configuration.

One group of prominent absorbers are periodically stacked metal insulator Fabry-Perot structures with lossy thin metals that are lithography-free and applicable to large areas and require rather few different types of materials to be deposited. Moreover, these structures exhibit extended bandwidth into mid-infrared (MIR) when the number of MI layers is increased. Not only do they eradicate the need for EBL but they also outperform most of the plasmonic absorbers. One of the most prominent results that gained attention for Fabry-Perot cavity absorbers was reported by Kats *et al*. in which nanometer thick anti-reflection coatings (ARC) resulted in absorption in a simple two-layer structure^12^. Several different metals and insulators were then utilized to get ultra-broadband perfect light absorption. W-Al_2_O_3_^13^, Cr-SiO_2_^14^, Ag-Si^15^, Ni/Ti-SiO_2_^16^, Au-PMMA-Cr^17^ and Cu-SiO_2_^18^ are some examples of these MI pairs to obtain perfect and broadband absorption. In MIMI absorbers, the bottom metal is optically thick and blocks the transmission of light and the middle metal layer is thin which partially transmits the light and couples it to the bottom MIM cavity where light is trapped and absorbed in the lossy thin metal in several back and forth bouncing off the metals. The highest bandwidth obtained in these structures is reported by Deng *et al*. where over 90 percent absorption is realized from 400 nm to 1400 nm utilizing Cr-Al_2_O_3_ MI pair^19^. However, in the afore-mentioned studies of MIMI absorbers, it is taken for granted that the bottom metal layer only functions as a reflector and could be any reflecting metal and the absorption mainly takes place in the middle thin metal. Though the latter statement is undeniably valid, the former statement is controversial due to the fact that the incident light on structure observes an equivalent impedance of the whole structure in which all layers play a role and the bottom metal is not an exception. In addition, the phase shift in the bottom metal layer contributes to the phase matching of reflected and incident lights and this in turn changes the reflection and transmission properties of light in such a low quality factor asymmetric Fabry-Perot cavity. There is a recent report in which multi thickness versions of MIMI absorbers are designed to obtain enhanced broadband absorption^20^.

In this study, we provide an analysis for the selection of each metal layer and we point out that the bottom reflector metal affects the absorption qualities that help us fabricate an MIMI absorber with the broadest bandwidth between previously reported MIMI absorbers, to the best of our knowledge to date. First, the simulation and analytic study are carried out to obtain optimized factors. Afterwards, the material choice for the middle thin metal layer is studied. After securing the lossiest metal, we have adapted the impedance transfer method where the equivalent impedance is matched to that impedance of air to attain the ideal permittivity values of a bottom metal.

## Calculation and Analysis

**Figure 1:**
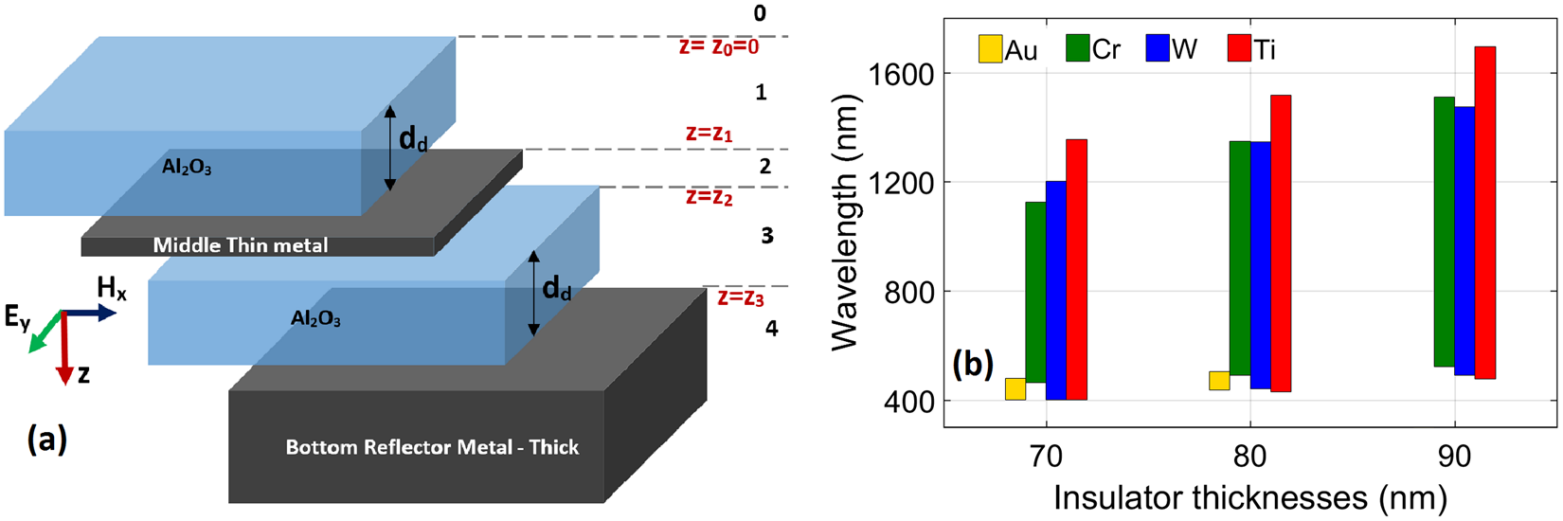
(**a**) Schematic of the optimized MIMI structure and (**b**) over 90 percent absorption window for four different double metal-Al_2_O_3_ stacks. The insets of Fig. 1 (**a**) illustrate field directions, the direction of propagation (TE) and layer and boundary numbers.

The schematic of the proposed optimum structure is illustrated in Fig. 1(a).

**Figure 2:**
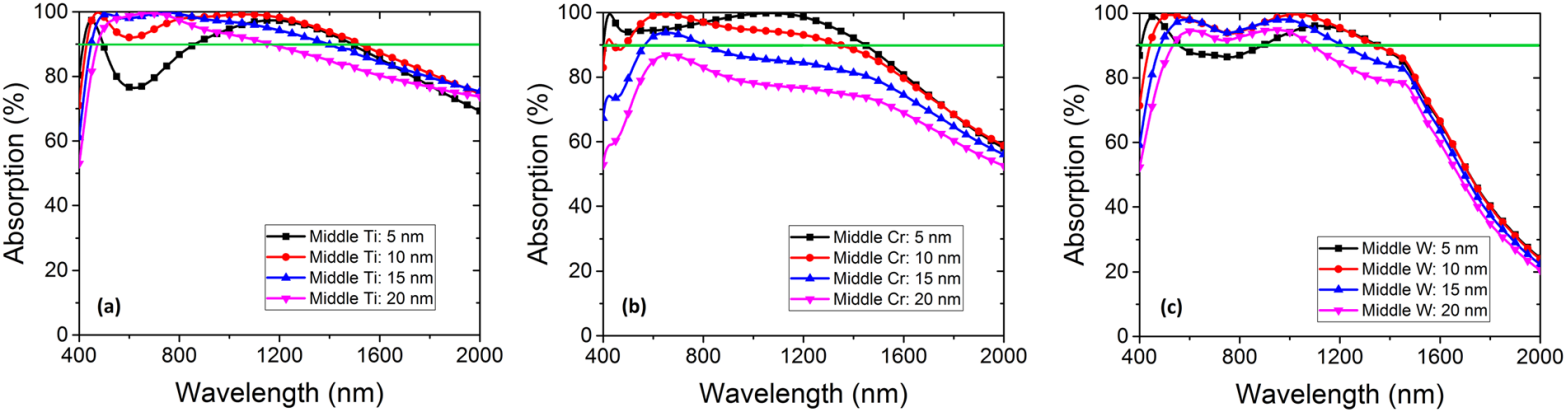
Absorption versus wavelength of double MI pairs (MIMI) with thick bottom metals and the same middle thin metal materials of 5 nm, 10 nm, 15 nm, and 20 nm thick (**a**) Ti, (**b**) Cr, and (**c**) W.

In order to design the absorber, in this section we discuss the optimization process of the thickness of the materials. In the following section we will scrutinize material choice process. Figure 1(b) illustrates the absorption over 90 percent calculated in double pairs (MIMI) of Au-Al_2_O_3_, Cr-Al_2_O_3_, W-Al_2_O_3_ and Ti-Al_2_O_3_. In each of these calculations, the configuration in Fig. 1(a) is employed and the middle thin metal and bottom reflector metal are the same. This calculation is repeated for three lossy metals and gold in order to provide a fair comparison. Figure 1(b) depicts over 90 percent absorption bandwidths for the mentioned absorbers for three different Al_2_O_3_ layer thicknesses (the thickness of top and sandwiched Al_2_O_3_ layers are equal in each calculation) where the bottom metal is selected to be optically thick and the middle thin metal is 10 nm. It is inferred from Fig. 2(a) that gold is not competent with lossy metals in perfect absorption. Besides, among different metals, Ti is the best metal of choice for perfect and broadband absorption. Moreover, the Al_2_O_3_ thicknesses of 70 and 90 nm suffer from extension into NIR and losing visible frequencies respectively. Therefore, 80 nm is the better choice of thickness for Al_2_O_3_ layers.

In order to calculate absorption, we have implemented transfer matrix method (TMM) for the structure to obtain results of Fig. 2(b). Since we have thick bottom metals in our designs, the transmission (*T*) vanishes and the absorption (*A*) reduces to *A* = 1 − *R* − *T* = 1 − *R* where *R* stands for reflection. In order to investigate TMM in the proposed four-layer structures, the notations depicted in Fig. 1(a) are used, with media 1 to 4 representing the device from top to bottom where a TE wave with x-polarized electric field is incident on sample. One can assume that the incident wave is TEM since we discuss normal incidence only. For each medium, *d*_*i*_, ${\gamma }_{i}={\alpha }_{i}+j{\beta }_{i}=j\omega \sqrt{{\mu }_{i}{\varepsilon }_{i}}$, *μ*_*i*_ and *ε*_*i*_(*i* = 0, 1, 2, 3, 4) represent the thickness, complex propagation constant, complex permeability and complex permittivity of layer while *ω* stands for angular frequency. Assuming that there is a solution with total forward and backward propagating electromagnetic wave for each medium of the form

1 $${\bar{E}}_{y,i}={B}_{i}{e}^{-{\gamma }_{i}z}+{A}_{i}{e}^{+{\gamma }_{i}z},{\bar{H}}_{x,i}=\frac{1}{j\omega {\mu }_{i}}\frac{\partial {\bar{E}}_{y,i}}{\partial z}$$

where *A*_*i*_ and *B*_*i*_ are constants. The boundary conditions enforce continuity of tangential components of electric and magnetic fields (${\bar{E}}_{y}$, ${\bar{H}}_{x}$) at all boundaries between each two consecutive adjacent media at *z*_0_, *z*_1_, *z*_2_ and *z*_3_.

2 $${\bar{E}}_{y,i}{|}_{z={z}_{i}}={\bar{E}}_{y,i+1}{|}_{z={z}_{i}},{\bar{H}}_{x,i}{|}_{z={z}_{i}}={\bar{H}}_{x,i+1}{|}_{z={z}_{i}}.$$

Using the boundary conditions, a general transfer matrix can be derived to correlate the solutions of adjacent media at boundaries such that,

3 $$(\begin{array}{c}{A}_{i}\\ {B}_{i}\end{array})={({M}_{i})}_{2\times 2}(\begin{array}{c}{A}_{i+1}\\ {B}_{i+1}\end{array})$$

4 $$({M}_{i})=(\begin{array}{cc}\frac{1}{2}[1+\frac{{\gamma }_{i+1}}{{\gamma }_{i}}\frac{{\mu }_{i}}{{\mu }_{i+1}}]{e}^{({\gamma }_{i+1}-{\gamma }_{i}){{\rm{z}}}_{i}} & \frac{1}{2}[1-\frac{{\gamma }_{i+1}}{{\gamma }_{i}}\frac{{\mu }_{i}}{{\mu }_{i+1}}]{e}^{-({\gamma }_{i+1}+{\gamma }_{i}){{\rm{z}}}_{i}}\\ \frac{1}{2}[1-\frac{{\gamma }_{i+1}}{{\gamma }_{i}}\frac{{\mu }_{i}}{{\mu }_{i+1}}]{e}^{({\gamma }_{i+1}+{\gamma }_{i}){{\rm{z}}}_{i}} & \frac{1}{2}[1+\frac{{\gamma }_{i+1}}{{\gamma }_{i}}\frac{{\mu }_{i}}{{\mu }_{i+1}}]{e}^{-({\gamma }_{i+1}-{\gamma }_{i}){{\rm{z}}}_{i}}\end{array}).$$

Thus, since the transmission is zero, the ratio of $R=|{A}_{0}/{B}_{0}|$ would lead to absorption (*A* = 1 − *R*) in the structure. One would need to solve the following equation,

5 $$(\begin{array}{c}{A}_{0}\\ {B}_{0}\end{array})=({M}_{0})({M}_{1})({M}_{2})({M}_{3})(\begin{array}{c}{A}_{4}\\ {B}_{4}\end{array})$$

assuming that *B*_4_ is equal to zero due to very thick bottom reflector metal. In other words, there is no backward propagating wave in medium 4. In order to solve this equation, it is assumed that the materials are non-magnetic^21^. The permittivity values of dielectric layers are determined to be ${\varepsilon }_{1}={\varepsilon }_{3}={\varepsilon }_{d}=1.7$ which is an acceptable value for extracted permittivity of our deposited Al_2_O_3_. Analogous to the simulations, ${\varepsilon }_{2}={\varepsilon }_{Ti}$ and ${\varepsilon }_{4}={\varepsilon }_{W}$ are defined with Palik model^22^.

## Results and Discussion

The optimal thicknesses for spacer and ARC dielectrics (i.e. the one sandwiched between metals and the one on top respectively) of the previous section (*d*_*d*_ = 80 nm) are used in the following simulations. The absorption results for three metals Ti, Cr and W in MIMI structure are illustrated in Fig. 2(a–c) where Au is left out due to weak absorption quality. In each panel, the absorption is depicted for four different thin middle metal thicknesses (i.e. 5, 10, 15 and 20 nm).

It can be inferred from the results presented in Fig. 2 that as expected from the results of Fig. 1(b), Ti shows superior performance. In addition, a 5 nm thin metal layer in all of the MIMI structures results in the presence of dips in the visible region below 90 percent, which is not appropriate. 15 and 20 nm thin metal layers result in reduced absorption bandwidth and peak. Thus, 10 nm of middle metal is the optimal thickness where the trade-off between the breadth and peaks of absorption are compensated reasonably.

**Figure 3:**
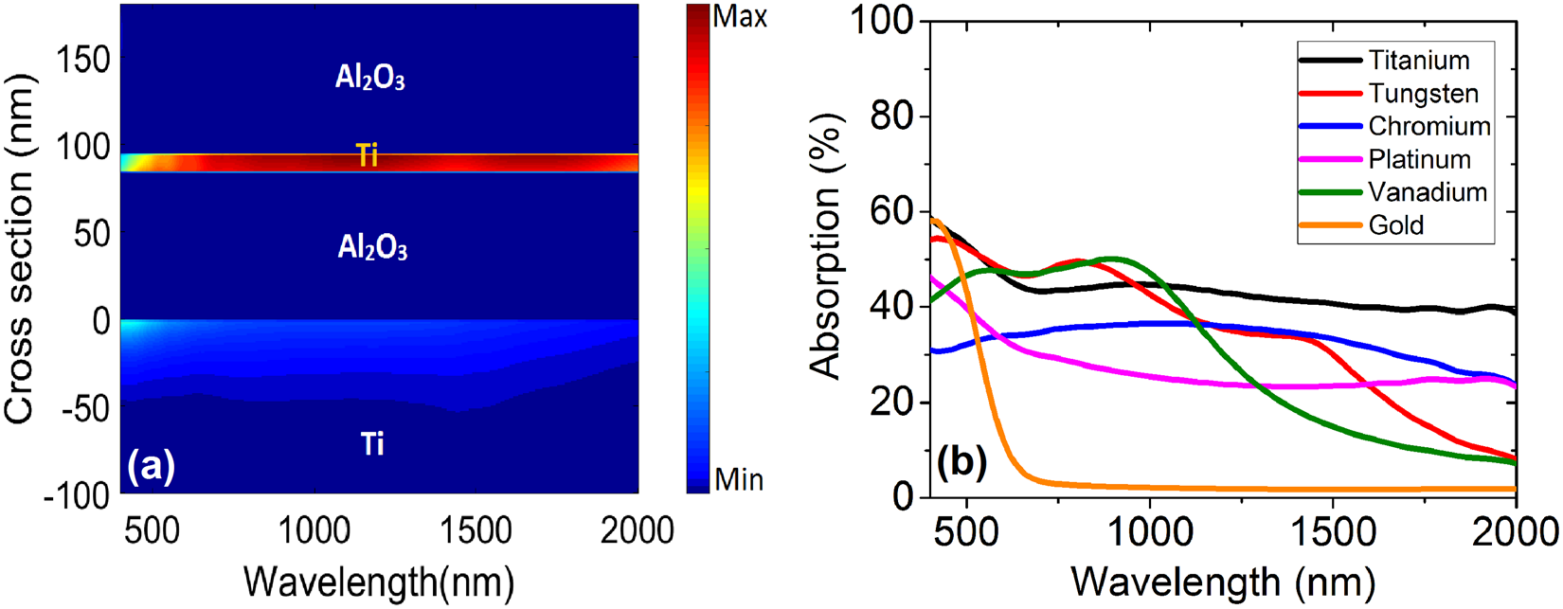
(**a**) Simulated contour plot of absorption in a cross-section of the MIMI sample with optimum parameters, (**b**) simulated absorption versus wavelength for some metals of infinite slab thickness.

In order to further elucidate the material choice and contribution of the layers, the lossy behavior of metals are considered. As visualized in Fig. 3(a), the thin metal layer possesses the most vital contribution to the absorption of the MIMI structure. This figure represents the simulated contour plot of absorption percentage in different layers of our proposed MIMI absorber. Our results also confirm that there is a contribution in the overall absorption from the bottom reflector metal that despite being small is not negligible. There are many lossy metals that can be chosen, but in order to achieve the best choice, absorption in infinite slabs of different metals are simulated for wavelengths running through 400 nm to 2000 nm. A commercial-grade simulator based on the finite-difference time-domain method was used to perform the calculations^23^.

Absorption in these slabs is illustrated in Fig. 3(b) for some lossy metals as well as gold in order to provide a comprehensive comparison between the absorption of thick metal layers. It is evident in this figure that Ti shows better overall performance in absorption and is, therefore, the best metal of choice for the thin lossy layer that is not proposed in the MIMI absorbers to the best of our knowledge.

In MIMI absorbers, the incident light is coupled into the system with the help of a top ARC layer with minimum reflection. Afterwards, it is partially absorbed in the thin metal layer and partially coupled into the cavity formed by the bottom MIM part of the structure. Inside this cavity, the light goes through several back and forth paths and since its intensity is reduced in the beginning, it cannot surpass the thin metal and get reflected to air. Thus, it gradually decays only inside the two metal layers due to the lossless nature of Al_2_O_3_. Moreover, Figs 2 and 3 also lead to the conclusion that the first part of absorption in lower wavelengths is mostly due to loss in bottom thick metal and the second one is mostly a result of absorption in thin middle metal layer. Taking Fig. 3(a) as an example, at 400 nm, the total amount of absorption in the bottom Ti metal and the middle thin Ti layers are 60 percent and 15 percent, respectively. At 1000 nm, on the other hand, these amounts are 25 percent and 72 percent respectively for bottom and middle Ti layers.

To gain further insight into how the perfect, broadband absorption is achieved by the MIMI structures, the impedance matching and ideal permittivity extraction is investigated in the following to highlight the choice of proper bottom reflector metal. The choice of metals for the bottom reflector has not been investigated much in papers, and it is deemed to be only a good reflector^19^. Quite contrarily, since all metals in the structure contribute to the equivalent impedance of the structure, they are a pivotal part of the structure in such a low quality factor cavity.

**Figure 4:**
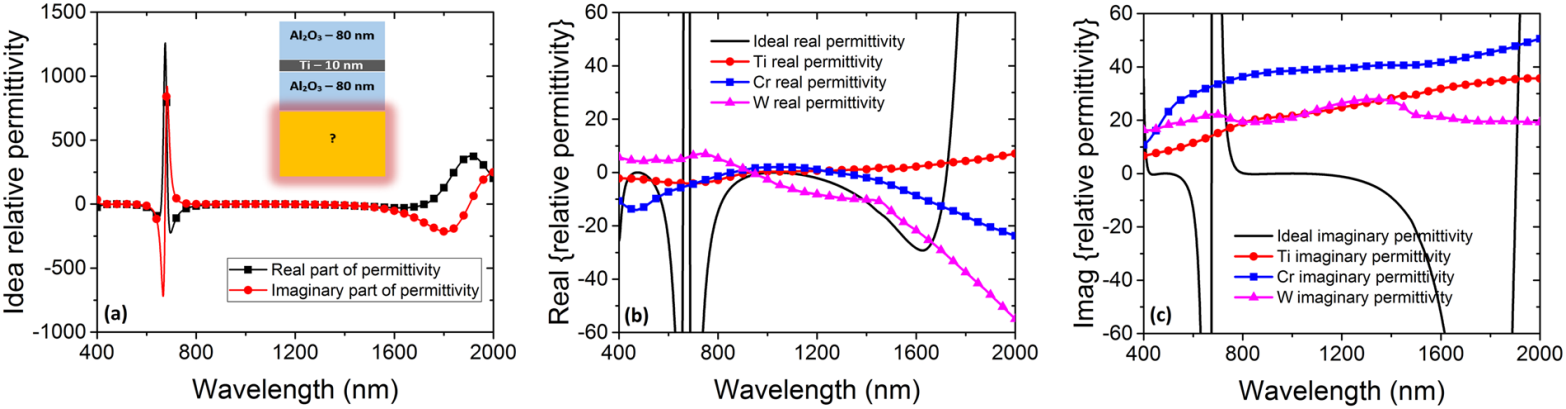
(**a**) Calculated ideal real and imaginary parts of permittivity for bottom reflector thick metal, (**b**) ideal real relative permittivity versus real parts of relative permittivity of Ti, Cr, and W and (**c**) ideal imaginary relative permittivity versus imaginary parts of relative permittivity of Ti, Cr, and W. The inset of Fig. 4 (**a**) shows the known and unknown calculation parameters.

Here, in order to retain perfect absorption, we investigate the choice of an optimum bottom metal for Ti thin middle metal with Al_2_O_3_ thicknesses set to 80 nm. First, we calculate ideal permittivity values for the bottom reflector metal and compare it to the existing metals. In other words, the calculations are carried out for an optimum MIMI structure with the bottom reflector metal set to be unknown and the IMI layers are 80 nm Al_2_O_3_, 10 nm Ti and 80 nm Al_2_O_3_ respectively. Therefore, by ideal permittivity values we try to find out what is the ideal material of choice for the bottom reflector layer. Afterwards, the ideal permittivity is compared with different metals and the closest possible choice is pointed out for broadband perfect absorption. The method used here is a calculation of the normalized impedance of the system to the impedance of free space and then setting it equal to one to obtain perfect impedance matching. The cited total, normalized effective impedance is calculated using the impedance transfer method derived by^18,19^

6 $${Z}_{T}=\frac{{A}_{1}+{A}_{2}{n}_{R}}{{B}_{1}+{B}_{2}{n}_{R}}$$

where

7 $${A}_{1}={n}_{d}{n}_{Ti}\,{\tan }^{2}({\phi }_{d})+{n}_{d}{n}_{Ti}{\phi }_{Ti}({{n}_{d}}^{2}+{{n}_{Ti}}^{2})-{{n}_{d}}^{2}{n}_{Ti},$$

8 $${A}_{2}=-j{{n}_{Ti}}^{2}{\phi }_{Ti}\,{\tan }^{2}({\phi }_{d})+j2{n}_{d}{n}_{Ti}\,\tan ({\phi }_{d})-{{n}_{d}}^{2}{\phi }_{Ti},$$

9 $${B}_{1}=-j{{n}_{d}}^{4}{\phi }_{Ti}\,{\tan }^{2}({\phi }_{d})+j2{{n}_{d}}^{3}{n}_{Ti}\,\tan ({\phi }_{d})+{\rm{j}}{{n}_{d}}^{2}{{n}_{Ti}}^{2}{\phi }_{Ti},$$

and

10 $${B}_{2}={{n}_{d}}^{2}{n}_{Ti}\,{\tan }^{2}({\phi }_{d})+{n}_{d}{\phi }_{Ti}({{n}_{d}}^{2}+{{n}_{Ti}}^{2})\,\tan ({\phi }_{d})-{{n}_{d}}^{2}{n}_{Ti}.$$

where ${\phi }_{i}=j{\gamma }_{i}{d}_{i}$ represents the phase shift coming from each layer and *γ* and *d* represent the complex propagation constant and thickness of layers, respectively. *n*_*R*_ stands for the complex refractive index of the bottom thick reflector material. In equations (6–10), *n*_*Ti*_ and *n*_*d*_ are the refractive index of the thin Ti layer and the Al_2_O_3_ layers, respectively. *φ*_*d*_ and *φ*_*Ti*_ are, respectively, the phase shifts for Al_2_O_3_ and Ti layers. It is worth reciting that the thicknesses of the two Al_2_O_3_ layers are equal. Therefore, the phase shift in each of these layers is equal and is represented with *φ*_*d*_. In the derivation of equation (6), it is assumed that ${\phi }_{Ti}=j{\gamma }_{Ti}{d}_{Ti}\ll 1$ due to the very thin layer of Ti (10 nm). It follows that $\tan ({\phi }_{Ti})\approx {\phi }_{Ti}$. Now, by applying *Z*_*T*_ = 1 condition on equation (6) and considering the optimum thicknesses and material parameters for the other three layers, we obtain the ideal refractive index *n*_*R*_ of the optically thick bottom layer. Figure 4(a) illustrates the ideal permittivity values versus wavelength calculated by solving equation for *n*_*R*_ and using the relation ${\varepsilon }_{rR}=\varepsilon \text{'}+j\varepsilon \text{'}\text{'}={{n}_{R}}^{2}$.

The main reason for the spike of the ideal permittivity values in Fig. 4(a) is the cavity resonance. In the vicinity of 600 nm, the wave impedance of the structure does not match that of the free space which is a signature of the cavity resonance, meaning that there must be a peak of reflection (or absorption dip) around this wavelength. This resonance depends on the materials and thicknesses of the layers in the cavity. Moreover, Fig. 4(b) and (c), respectively, compare the real and imaginary parts of relative permittivity for Ti, Cr, and W bottom reflector metals where the middle metal is Ti (inset of Fig. 4(a)). From Fig. 4(b) and (c) it is observed that the permittivity of W is closer to the ideal values overall in terms of both real and imaginary parts, i.e. the real and imaginary parts of relative permittivity for other metals deviate drastically from the ideal case. Our comparison also shows that W is the closest possible metal to the ideal values between all other metals when the middle metal is Ti. This is interesting due to the fact that Ti as the thin metal layer does not yield the best performance with itself as the bottom reflector metal. Moreover, Fig. 4(b) and (c) show that the dip in the absorption results observed in Fig. 2(a) are a result of mismatch in the permittivity of W with the ideal values in the vicinity of 600 nm. Since there is a finite choice of materials, it is not feasible to find a natural material that has the same spike in its permittivity as the ideal values in Fig. 4(a). Therefore, this peak is visible in the experimental and simulation results of the structure in the following paragraphs as well as in all three panels of Fig. 2. In other words, if we had an imaginary material with spike in its permittivity values analogous to the ideal values, the reflectance peak of the results would be compensated and no dip in absorption results would have been detected.

**Figure 5:**
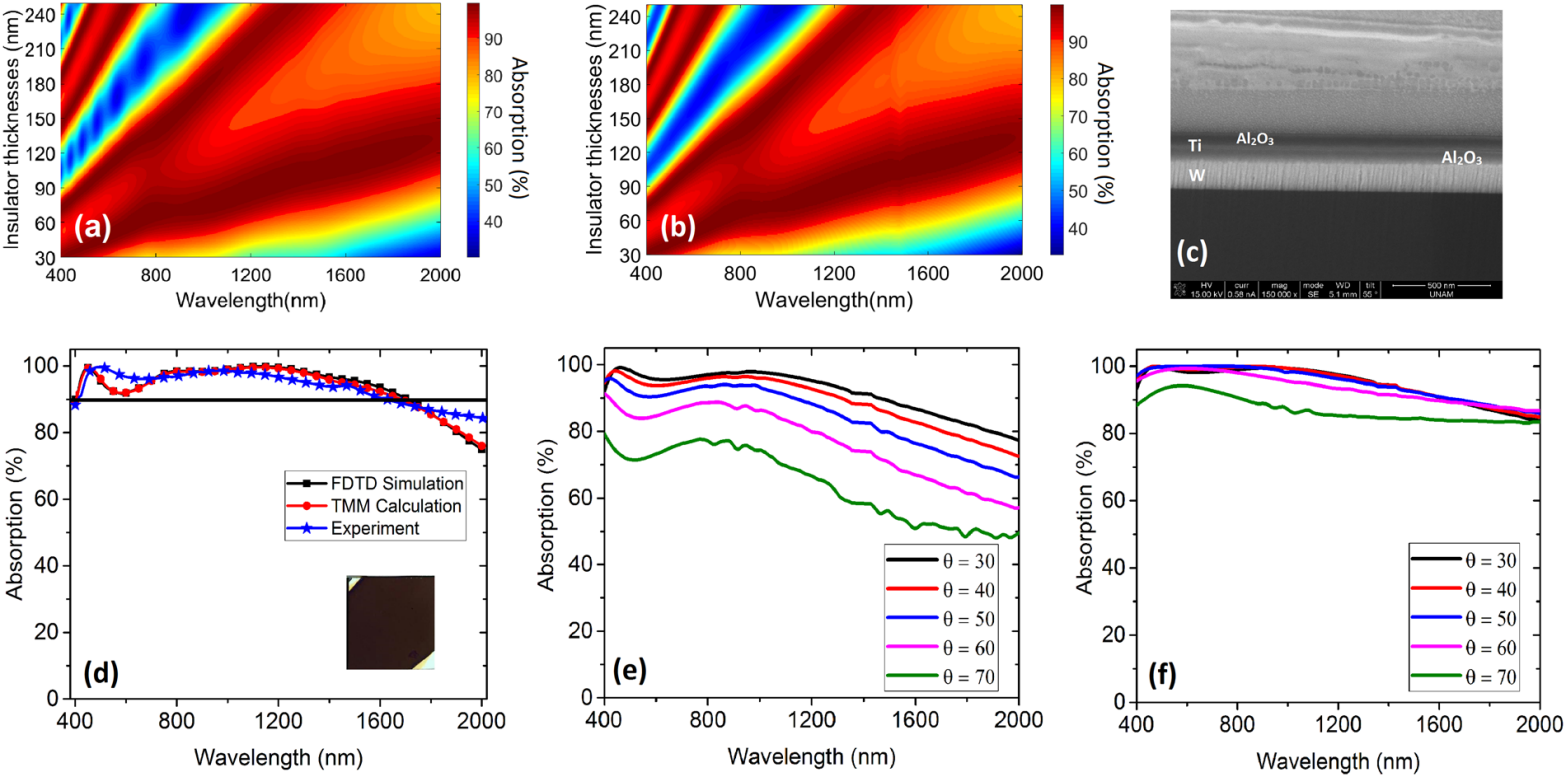
(**a**) Simulated absorption versus the wavelength and dielectric thicknesses for the optimum MIMI sample with *d*_*d*_ = 80 nm, *d*_*Ti*_ = 10 nm, (**b**) calculated counterpart of Fig. 5 (**a**), (**c**) cross section FIB image of the fabricated sample, (**d**) measured, simulated, and calculated (TMM) absorption at normal incidence, absorption of fabricated sample (**e**) measured at different incidence angles (*θ* degrees) for TE polarization and (**f**) measured at different incidence angles (*θ* degrees) for TM polarization. The inset of Fig. 5 (**d**) shows the sample photo.

In order to glance at the optimum structure once more, Fig. 5(a) and (b) reveal the contour plot of absorption versus dielectric (Al_2_O_3_) thicknesses (*d*_*d*_) and wavelength for the final MIMI structure calculated with FDTD simulation and TMM method respectively. In these figures, the thickness of the middle thin Ti metal layer (*d*_*Ti*_) is 10 nm and the bottom reflector metal is optically thick W. The contour plot coloring is modified such that the red regions demonstrate the absorption of over 90 percent, which makes it easy to visualize the bandwidth of the perfect absorption. It should be pointed out that the thickness *d*_*d*_ is set equal in both spacer and ARC dielectrics (i.e. the one sandwiched between metals and the one on top, respectively). These results illustrate the absorption capability of the stacks as a function of dielectric thickness for *d*_*d*_ values of 30 nm to 250 nm as well as for a thin Ti layer thickness of 10 nm (Fig. 5(a)). In our simulations, we use the Palik model for Ti and W^22^ and the permittivity values of Al_2_O_3_ are extracted using J. A. Woollam VASE Ellipsometer and imported to Lumerical FDTD as sampled data. The simulation is carried out in 2D using Lumerical FDTD. The TMM contour counterpart of simulation is provided in Fig. 5(b) using equations (4) and (5).

After completing the material optimization in the previous parts and obtaining the optimal design, the MIMI absorber was fabricated and characterized. The FIB cross section image of the fabricated sample is shown in Fig. 5(c). The experimental normal reflection measurement of the sample as well as TMM and simulation results for optimized thicknesses (*d*_*d*_ = 80 nm and *d*_*Ti*_ = 10 nm) are put forth in Fig. 5(d). The simulated and calculated absorption as well as the experimental results are provided together for the mentioned thicknesses for the sake of comparison. The simulation, calculation, and experimental absorption results demonstrate 400 nm to 1705 nm, 400 nm to 1685 nm and 400 nm to 1642 nm, respectively. The absorption is retained over 90 percent at normal incidence onto the MIMI sample from 400 nm to 1642 nm which is close to the numerical results estimated by simulation and TMM. The response for different incident angles (*θ*) on the sample for TE and TM polarizations are provided in Fig. 5(e) and (f), respectively, which emphasize the fact that the sample has quite well absorption performance in angled illumination as well. The fabricated optimum sample deviates a little from the simulation and calculation results. This is attributed to the exact thickness of layers which is found to be 82 nm for Al_2_O_3_ layers and 9.5 nm for Ti. The thickness for dielectric and Ti layers are calculated from the results of ellipsometer and FIB cross section presented in Fig. 5(c).

One of the most important applications for such a simple and high-performance structure that contains refractory metals is in thermal emitters due to the fact that they are very resistant to increased heat^19^. Furthermore, this structure possesses very high absorption concentrated in an ultra-thin metal layer, i.e. Ti. This gives rise to an outlook of this structure to be an outstanding candidate for photoelectric effect. Multilayer photodetectors operating in near-infrared (NIR) region are the reported alternatives for bulk NIR absorbers and PN photodetectors that are only functional at cryogenic temperatures. Though these designs are engineered with plasmonic absorption^24,25^, a better alternative would be MIMI structures that manage to confine light more effectively in thin layers while achieving this with simple large area multilayer processes.

We have experimentally demonstrated a perfect absorber that retains absorption over 90 percent between 400 nm and 1640 nm which is fabricated using lithography-free and large scale compatible methods. The choice of each layer is studied and optimized with simulations and numerical calculations employing transfer matrix and FDTD methods. The significance of metal layers and their contribution in overall absorption is discussed in detail. Moreover, impedance matching to air and loss of metals are proposed as a versatile tool to obtain the most appropriate metallic layer choices for MIMI broadband perfect absorbers. These absorbers are potential candidates to be tailored in applications ranging from thermal imaging and emitters to photovoltaics and photodetectors of next generation devices.

## Methods

The fabrication starts with a silicon wafer with unintentional doping cleaned with acetone and isopropanol. Then, a 150 nm deposition of W is carried out in the VAKSIS sputtering system in a base pressure of 1e-6 Torr, Ar flow of 30 sccm, DC power of 150 W and a deposition pressure of 20 mTorr. Afterwards, 80 nm Al_2_O_3_ is deposited using Cambridge Nanotech. Inc. Savannah-S100 atomic layer deposition (ALD) system. The deposition is realized using Trimethylaluminum and milli-Q water precursors repeated 820 cycles in an N_2_ flow of 20 sccm. Following this step was the deposition of 10 nm Ti in the VAKSIS thermal evaporator system in a base pressure of 1–3 × 10^−6^ Torr. The last step was identical to the ALD deposition mentioned earlier and all the parameters were kept the same. For the characterization, the reflection of sample was measured in Bruker Vertex 70 v FTIR and the angled reflection was measured using J. A. Woollam VASE Ellipsometer.
